# The hematopoietic regulator, ELF-1, enhances the transcriptional response to Interferon-β of the *OAS1* anti-viral gene

**DOI:** 10.1038/srep17497

**Published:** 2015-12-08

**Authors:** Steven Larsen, Shota Kawamoto, Sei-ichi Tanuma, Fumiaki Uchiumi

**Affiliations:** 1 Research Center for RNA Science, RIST, Tokyo University of Science, Noda, Chiba, Japan; 2 Department of Gene Regulation, Faculty of Pharmaceutical Sciences, Tokyo University of Science, Noda, Chiba, Japan; 3 Department of Biochemistry, Faculty of Pharmaceutical Sciences, Tokyo University of Science, Noda, Chiba, Japan

## Abstract

Interferon (IFN) therapy is effective in treating cancers, haematological and virus induced diseases. The classical Jak/Stat pathway of IFN signal transduction leading to changes in transcriptional activity is well established but alone does not explain the whole spectrum of cellular responses to IFN. Gene promoters contain *cis*-acting sequences that allow precise and contextual binding of transcription factors, which control gene expression. Using the transcriptional response to IFN as a starting point we report a high frequency of tandem GGAA motifs in the proximal promoters of Interferon stimulated genes, suggesting a key regulatory action. Utilizing the well-characterized anti-viral gene, *OAS1*, as an example Interferon stimulated gene promoter containing such a duplicated GGAA motif, we have demonstrated a regulatory role of this promoter in response to IFN by mutation analysis. Furthermore, we identified ELF-1 as a direct binding factor at this motif. Additionally, recruitment of RB1 and SP1 factors to the promoter following IFN stimulation is shown. ELF-1 overexpression enhanced and knockdown of ELF-1 inhibited full activation of *OAS1* by IFN stimulation. Collectively, ELF-1 binds an important duplicated GGAA cis-acting element at the *OAS1* promoter and in cooperation with RB1 and SP1 recruitment contributes to regulation in response to IFN stimulation.

The regulation of gene expression by extracellular signals requires the interplay between multiple transcription factors that selectively bind to gene promoters with spatial and temporal precision. Gene deregulation is a common factor of human diseases, including cancers and immune disorders. From the study of cellular differentiation in macrophages, essential duplicated GGAA-motifs which are recognized by various transcription factors including ETS family proteins^1^, were discovered in the promoters of human *PARG*^2^ and *IGHMBP2* genes^3^. Moreover, the duplicated GGAA motifs are frequently found in immune-function associated promoters including human *ISG15* and *CD40* genes^4^. These observations suggested that duplicated GGAA-motifs are common *cis*-acting elements that respond to activation of immune responses.

The biological features of the IFN response include anti-viral activity^5^, inhibition of cell proliferation, induction of differentiation and significant anti-tumor activity^6^,^7^. IFNs mediate these effects on target cells through induction of transcription for several hundred genes, known as the interferon-stimulated genes (ISGs)^8^. Cell type specific responses to IFNs have been observed, particularly in cell subtypes of the immune system^9^,^10^. Therapeutic use of IFNs include chronic hepatitis^11^, cancers and multiple sclerosis^12^. Due to the numerous and serious side-effects associated with IFN treatment, there is a urgent need for alternative narrow-spectrum IFN-derived therapeutics^13^. An understanding of the complex pathways induced in response to IFN-stimulation in cell sub-types and under specific cell conditions^14^ are crucial to improve the efficacy and patient uptake rate of IFN- derived therapeutics.

Here, for the first time, we report that ELF-1 recognizes and directly binds to a duplicated GGAA motif proximal to the transcription start site of the 2′-5′-Oligoadenylate synthetase 1 (*OAS1)* gene. Moreover, we demonstrated that ELF-1 enhances *OAS1* transcription and the transcriptional response to IFN with co-recruitment of SP1 and RB1. OAS1 is one of the most extensively characterized enzymes induced by IFNs, which is crucial for an effective anti-viral response. The OAS1 enzyme responds to double-stranded RNA by catalyzing the reaction of ATP to 2′-5′-oligoadenylates, which in turn activates latent ribonuclease (RNaseL)^15^,^16^, resulting in degradation of viral and cellular RNA and inhibition of protein synthesis^17^. The ETS transcription factor, E74-Like Factor 1 (ELF-1), is a key transcription factor in the regulation of genes that are involved in hematopoiesis and angiogenesis^18^,^19^,^20^,^21^,^22^. Regulation of ELF-1 occurs mainly through post-translational modifications involving O-glycosylation and phosphorylation by protein kinase C^23^ and protein interactions^24^. Our results have implications for development of novel IFN-based cancer therapies, such as artificially controlled ELF-1 expression and gene therapy.

## Results

### High frequency of duplicated GGAA motifs in the promoters of human ISGs

IFNs mediate their effects on target cells through the induction of several hundreds of genes, collectively described as ISGs. Several duplicated GGAA motifs were found in close proximity to the TSSs of several ISGs^25^, thus we further investigated the prevalence of these motifs in a wider selection of human ISGs. From the computer assisted analysis as described in Methods, we discovered that duplicated GGAA motifs (GGAA motifs with spacers of between 0 and 10 bp are reported) are over-represented in the majority of promoter regions immediately upstream of ISGs (81%). For comparison, the promoters of randomly selected genes (51.7%) and random humanized DNA sequences of the same length (25%) were similarly analyzed (Table 1).

### A duplicated GGAA motif in the human *OAS1* promoter is required for effective IFN-mediated activation

**Figure 1:**
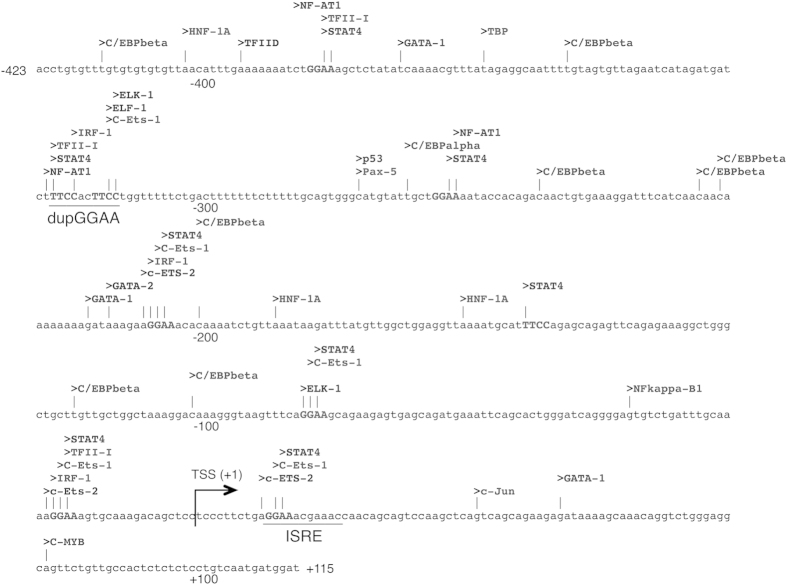
Putative transcription factor binding sites in the human *OAS1* promoter sequence. The binding sites were identified from the TRANSFAC database using PROMO virtual laboratory (http://alggen.lsi.upc.es/). The transcription start site (TSS; +1) is indicated by an arrow and the duplicated GGAA motif is highlighted.

**Figure 2:**
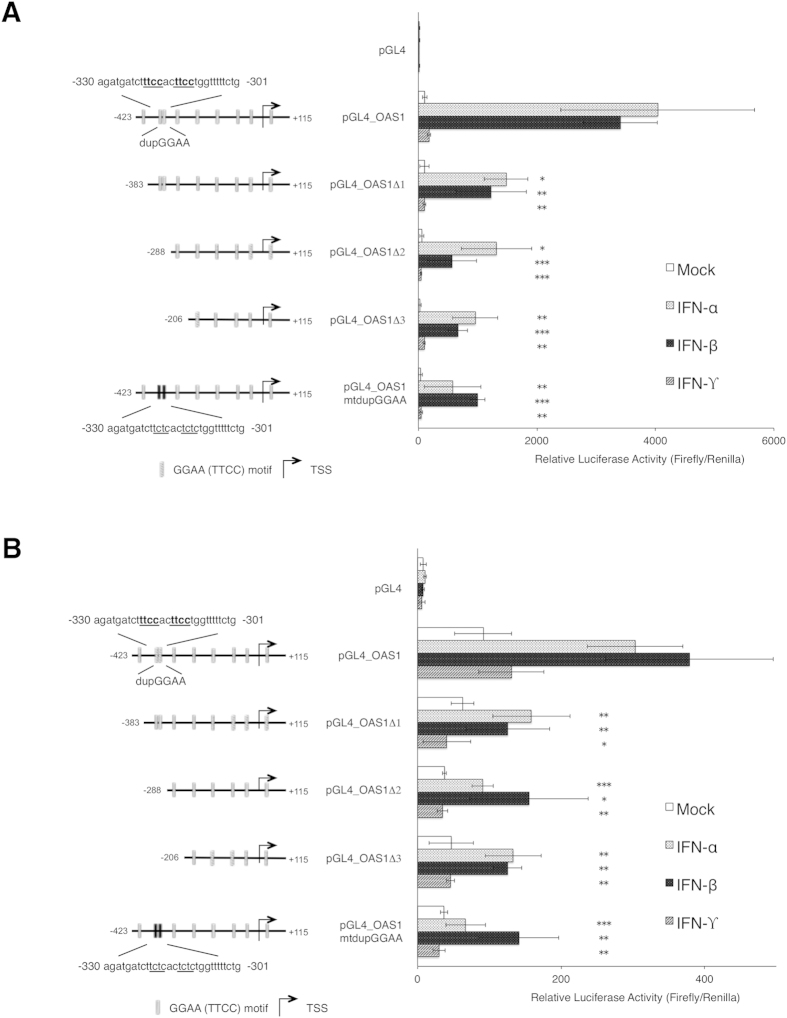
Role of duplicated-GGAA motif in IFN-mediated activation of the *OAS1* promoter. Site-directed mutagenesis of the wild type *OAS1* promoter containing pGL4_OAS1 *Luc* reporter plasmid was used as a template for site directed mutagenesis to create a *Luc* reporter plasmid with a disrupted duplicated GGAA motif, designated pGL4_mtdupGGAA. These *OAS1* reporter plasmids were transfected into HeLa S3 (**A**) or Jurkat (**B**) cells and 18 h later treated with IFN-α, -β, or -γ (100, 20 and 50 ng/ml, respectively) for 5 h, followed by *Luc* assay. Luc activity is shown relative to the expression of a *Renilla* luciferase control vector and error bars represent SD from three independent transfections. Comparisons between IFN-stimulated pGL4_OAS1 transfectants and either deletion or mutant dupGGAA were made by the student’s t-test; *p < 0.1; **p < 0.05; ***p < 0.01.

The *OAS1* gene is an important ISG encoding an enzyme with essential functions in anti-viral defense^26^. Even though an Interferon-stimulated response element (ISRE) is essential for the *OAS1* gene to respond to IFN but alone it is not responsible for full activation^27^. Therefore, we focused on the role of duplicated GGAA motifs in regulation of ISG induction by IFNs, analyzing the contribution of this motif to *OAS1* promoter activation. As depicted in Fig. 1, we have isolated a 541 bp region surrounding the reported TSS (accession number, NM_016816.2), which responds well to IFN stimulation (5 h), and examined its activity by *Luciferase* (*Luc*) reporter assay with serial deletion constructs of this region (Fig. 2). Deletion of the region containing the duplicated GGAA motif (−423 to −288) from the pGL4_OAS1 construct impaired the IFN-mediated promoter activation in both HeLa S3 and Jurkat cells. *Cis*-acting elements, such as in the promoters of ISGs are also responsible, at least in part, for the differential transcription profiles in response to IFN stimulation and hence the pleiotropic effects of IFNs^28^. Therefore, we speculate that the response may additionally originate from other GGAA (TTCC) containing elements, such as binding sites for STAT4, IRF-1 and ETS-family factors. The above result suggests that the deleted sequence from nucleotide −423 to −288 contains *cis*-acting element in the response to IFN-β and -γ. In order to determine the contribution of the duplicated GGAA motif to activation of the *OAS1* promoter, the motif in the 541 bp region was disrupted to make the pGL4_OAS1mtdupGGAA construct (Fig. 2). This substitution comparatively inhibited IFN-induced promoter activation, indicating that the duplicated GGAA motif (−326 to −307; 5′-gatctttccacttcctggtt-3′) is required for full promoter activation following IFN-treatment.

### Sequence specific DNA-binding complexes at a duplicated GGAA motif in the *OAS1* promoter

**Figure 3:**
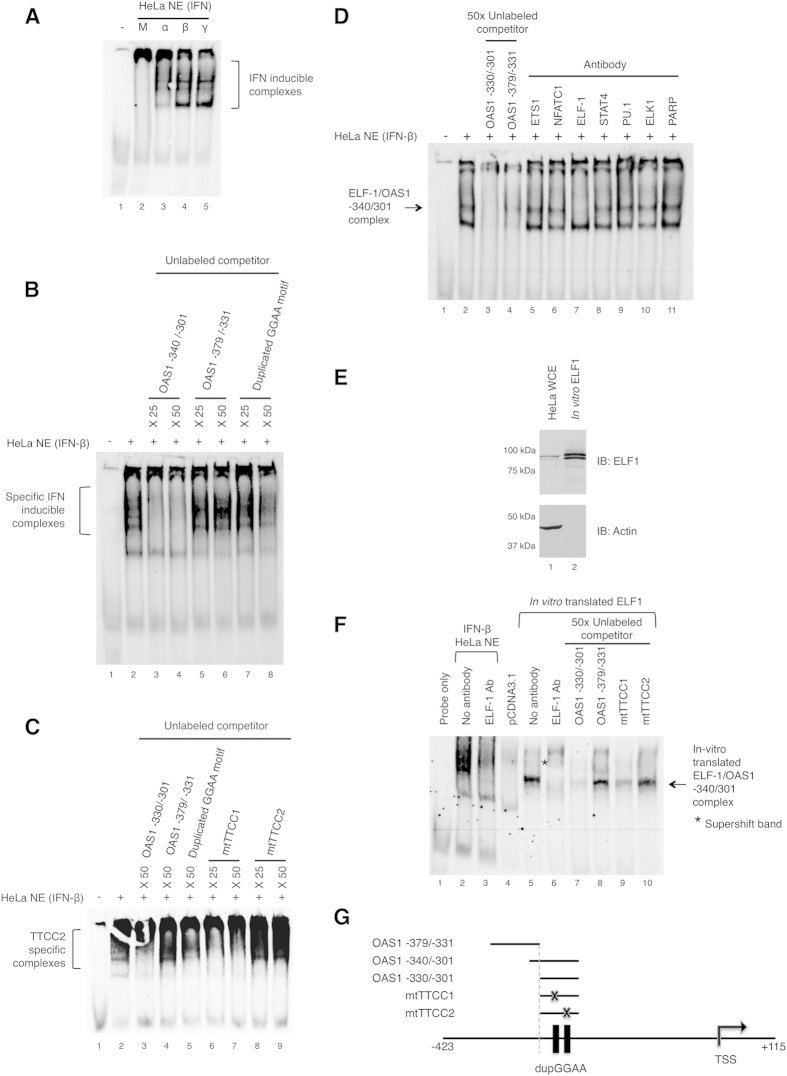
Formation of specific DNA-protein complexes at the *OAS1* promoter in response to IFNs. Identification of an ELF-1 complex(s) that specifically binds to the duplicated GGAA motif in the *OAS1* promoter. (**A**) Nuclear extracts derived from HeLa S3 cells, which were either treated with 100 ng/ml IFN-α, 20 ng/ml -β or 50 ng/ml -γ for 5 h (lanes 3, 4 and 5, respectively) or mock stimulated (lane 2), were subjected to EMSA with 3′-end DIG labeled probe, human *OAS1* −340/−301 (Table 2). (**B**,**C**) The specificity of binding complexes for GGAA motifs were determined by competition assays using unlabeled specific, non-specific competitors and mutated probes (Table 2). The molar excess of unlabeled competitor DNA duplex is either indicated by ×25 or ×50. (**D**) Supershift-EMSA analysis was performed with antibodies targeting ETS1, NFATC1, ELF-1, STAT4, PU.1, ELK1 and PARP (lanes 5–11, respectively) included in the binding reaction. An arrow indicates the position of the ELF-1 specific band. (**E**) Western blot showing the *in vitro* transcription/translated ELF-1 product from pcDNA3.1_ELF-1 plasmid (lane 2) and endogenous ELF-1 from HeLa S3 cell whole cell extract (lane 1). (**F**) EMSA analysis was performed with *in vitro* translated ELF-1 (lanes 5–10), lane 4 shows the binding reaction with the equivalent *in vitro* translation reaction product from the pcDNA3.1 empty vector; for comparison, binding reactions with IFN-β stimulated HeLa S3 nuclear extract (5 h) are shown (lanes 2 and 3). ELF-1 antibody was included in the binding reaction for lanes 3 and 6, competition with unlabeled specific, non-specific and mutated duplexes are shown in lanes 7–10: OAS1 −330/−301, specific; OAS1 −379/−331, non-specific; mtTTCC1, specific with mutation; and mtTTCC2, specific with mutation. An asterisk indicates the ELF-1 supershifted band. (**G**) Schematic showing location of the various probes listed in Table 2 and used in this figure.

To identify protein-DNA interactions at the duplicated GGAA motif (−326 to −307) in the *OAS1* promoter, we performed competition and supershift EMSAs with nuclear extracts prepared from IFN-stimulated (5 h) HeLa S3 cells. Specific protein-DNA interactions occurred at −340 to −301 of the *OAS1* promoter (OAS1 −340/−301) with nuclear extracts from IFN-α, -β and -γ HeLa S3 cells (Fig. 3A). In contrast to the promoter activation shown in Fig. 2, protein-DNA complexes were detected with the labeled OAS1 −340/−301 probe in binding reactions containing nuclear extracts from IFN-α and -β treated cells. This apparent difference could be the result of differences in transcriptional activation and response between IFN-α and -β. It is possible that these differences were reflected in the nuclear extracts used for EMSA assay. Additionally, fully quantitative comparisons between different nuclear extract preparations are difficult, even though prepared simultaneously. While a lot of useful information can be gained from *in vitro* EMSA, such experiments may not always reflect the precise temporal and spatial distribution of transcription factors in cells. Inclusion of the unlabeled specific competitor duplex in the binding reaction prevented formation of several shifted bands, demonstrating specificity of these interactions (Fig. 3B, lanes 2 to 4), whereas no competition was observed with the unlabeled adjacent sequence (OAS1 −379/−331; Fig. 3B, lanes 5 and 6). Although higher molar excess was required, a randomly generated synthetic duplicated GGAA motif containing unlabeled competitor inhibited formation of the same complexes (Fig. 3B, lanes 7 and 8). A competition assay using mutated probes identified only the second GGAA motif (intact in mtTTCC1) as being essential for formation of the DNA-protein complexes but not the first GGAA motif (intact in mtTTCC2, Fig. 3C). These experiments indicate that the second GGAA motif, 5′-acTTCCtg-3′, in the *OAS1* promoter is essential for binding of IFN induced factors.

### ELF-1 is an IFN-inducible transcription factor in HeLa S3 cells

As shown in Fig. 1, *in silico* analysis suggested several putative transcription factors, including ELK-1, ELF-1 and c-ETS-1, which may bind to the second GGAA motif. Therefore, we assessed the possible DNA-protein interactions by EMSA supershift with the relevant antibodies (Fig. 3D). Only the inclusion of an antibody targeting ELF-1 but not other antibodies in the binding reaction disrupted the formation of the GGAA specific binding complex (Fig. 3D). Thus, either ELF-1 or an ELF-1-containing complex present in the IFN-β treated (5 h) HeLa S3 cell nuclear extract can either directly or indirectly interact with the duplicated GGAA motif at the *OAS1* gene promoter. Additionally, a mobility shift band, representing ELF-1/*OAS1* −340/−301 complex (Fig. 3F, lane 5) was generated with *in vitro* translated ELF-1 (Fig. 3E), which was not observed with the translation reaction from empty expression vector (Fig. 3F, lane 4). This complex was super-shifted by an anti-ELF-1 antibody (Fig. 3F, lane 6) and was only competed out with a specific unlabeled competitor duplex (Fig. 3F, lanes 7 and 8).

Consistent with the results in Fig. 3C, the competition analysis using *in vitro* translated ELF-1 also demonstrated specificity of binding to the second GGAA motif (Fig. 3F, lanes 9 and 10). Taken together, these results demonstrate the ability of ELF-1 to form a direct physical interaction with the second GGAA motif in the *OAS1* promoter, which is in close proximity to the TSS. Additionally, a band of slightly faster mobility was seen with HeLa S3 nuclear extract when compared to *in vitro* translated ELF-1 protein (Fig. 3F, lanes 2 and 5, respectively), signifying that additional factors in the nuclear extract may contribute to the ELF-1/GGAA complex.

**Figure 4:**
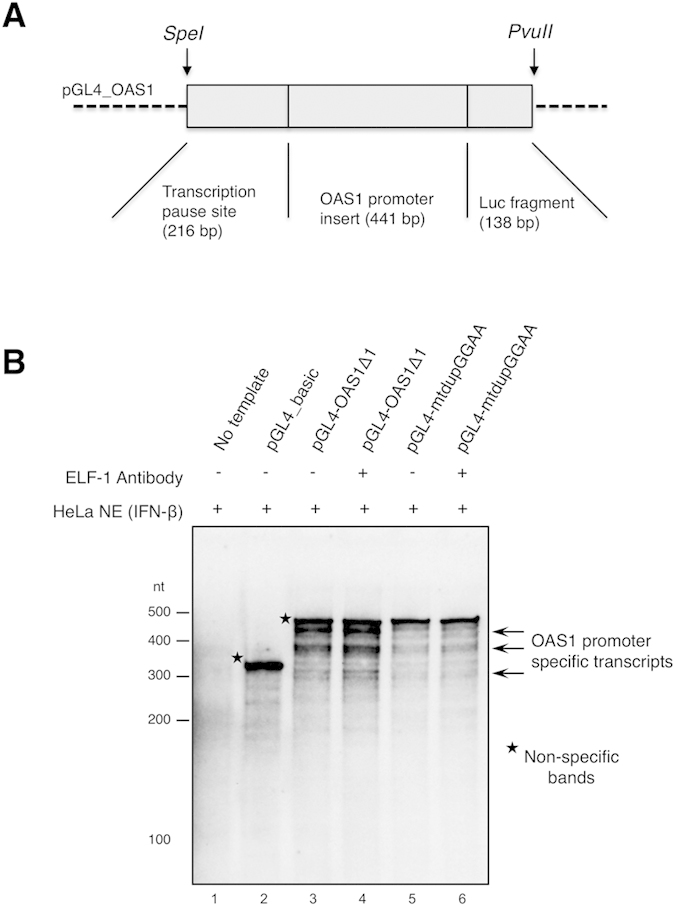
Determination of *de novo* RNA transcripts by non-radioisotope *in vitro* transcription assay (NITRA). (**A**) Diagram depicting the genetic arrangement of the *Spe*I*/Pvu*II fragments derived from OAS1 containing pGL4 plasmids, which include a transcription pause site, the promoter or mutated promoter insert and a fragment of the luciferase open reading frame. (**B**) NITRA indicates initiation of transcription from *Spe*I*/Pvu*II fragments of the pGL4_OAS1 or pGL4_OAS1mtdupGGAA plasmids. Templates were incubated with HeLa S3 nuclear extracts (HeLa NE), treated with IFN-β for 5 h, and then incubated in the presence of DIG-labeled dUTP, the *de novo* RNA transcripts were separated by denaturing PAGE and DIG-labeled RNAs were detected. An antibody targeting ELF-1 was included in the binding reactions (lanes 4 and 6). Non-specific bands (asterisks) originate from the ends of the template, which serve as an internal control.

We developed a sensitive non-radioisotope *in vitro* transcription run-off assay (NITRA) to show initiation of transcription from various *cis*-acting regulatory elements. Here, the sequence requirements for transcription from the *OAS1* promoter was analyzed by NITRA using nuclear extract from IFN-β treated HeLa S3 cells as a source of active transcription factors (Fig. 4). Disruption of the duplicated GGAA motif drastically reduced initiation of transcription from *Spe*I*/Pvu*II fragments of pGL4_OAS1 and pGL4_OAS1mtdupGGAA (Fig. 4A,B, compare lane 3 with 5). Although it was expected that the ELF-1 antibody would interfere with transcription from the *OAS1* promoter, the absence of the ELF-1 binding site affected transcription efficiency. Multiple transcripts were detectable, being consistent with previous reports of multiple transcription start sites in ISGs and TATA-less promoters^29^. Taken together these results show that the ELF-1 binding site is important for initiation of transcription, in which ELF-1 binding to the upstream duplicated GGAA motif in the *OAS1* promoter is strongly implicated.

### Induction of ELF-1 enhances *OAS1* transcription

**Figure 5:**
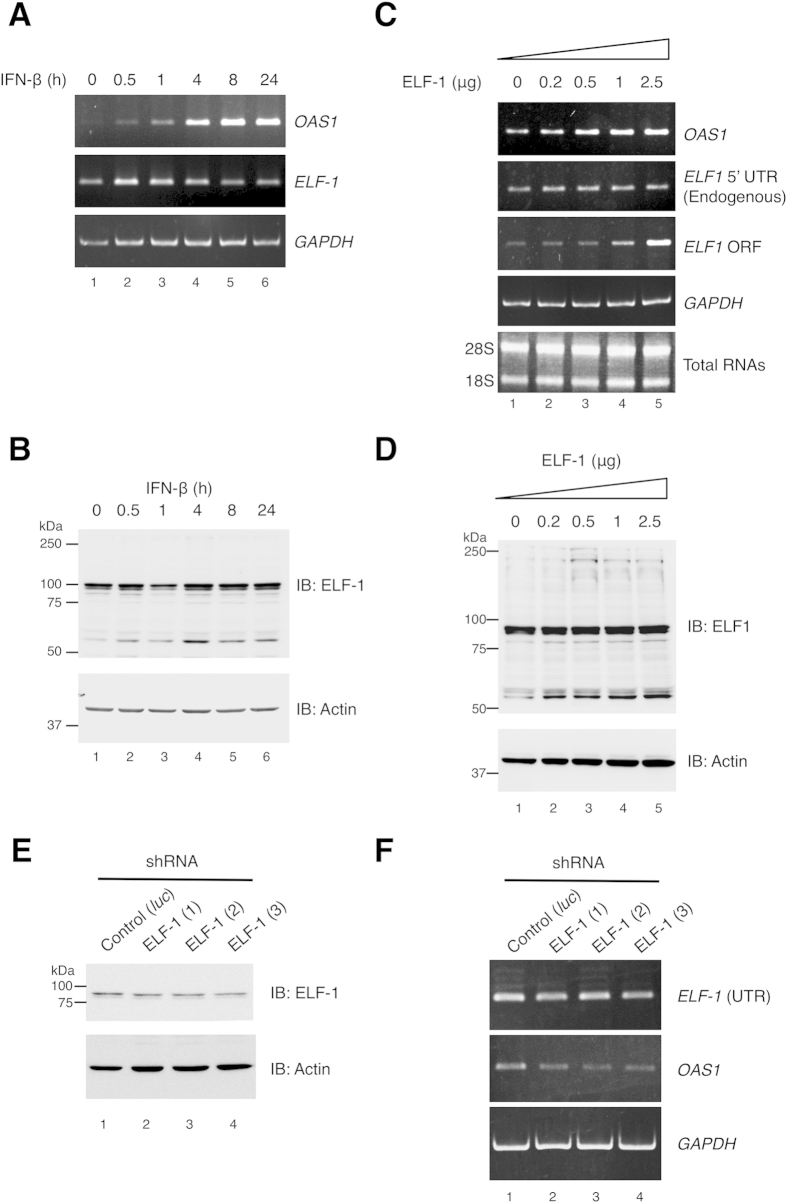
Effect of ELF-1 on *OAS1* gene expression and induction by IFN-β. (**A**) RT-PCR and (**B**) Western blot analysis was performed for detection of *OAS1*, *ELF-1* and *GAPDH* transcripts and detection of ELF-1 and actin protein levels, from HeLa S3 cells treated with IFN-β for the indicated time periods (0, 0.5 1, 4, 8 and 24 h), total RNAs are shown as an additional loading control. (**C**) RT-PCR and Western blot analyses of HeLa S3 cells after ectopic expression of ELF-1. HeLa S3 cells were transfected with increasing amounts of either pcDNA3.1_ELF-1 expression plasmids and harvested at 24 h post-transfection. Empty pcDNA3.1 plasmid was used to equilibrate the total amount of transfected DNA. PCR amplification of cDNA was carried out with the indicated primer sets (Supplementary Table). (**E**) Western blotting showing ELF-1 protein levels 72 hours after transfection of a control and three different ELF-1 targeting shRNA plasmids. (**F**) shRNA mediated knockdown of ELF-1 inhibits IFN-mediated *OAS1* gene expression. Semi-quantitative RT-PCR of HeLa S3 cells expressing three different ELF-1 shRNAs targeting ELF-1 are shown; Control (*luc*) indicates transfection with luciferase targeting shRNA as a control. RT-PCR and Western blot data show representative results from at least 3 and 2 experiments, respectively.

When HeLa S3 cells were stimulated with IFN-β from 0 to 24 h, rapid induction of *ELF-1* transcription was seen within 30 min of stimulation (Fig. 5A, lane 2). Notably, the IFN-β induced transactivation of *ELF-1* preceded accumulation of *OAS1* mRNA, which reached a plateau at 8 h post-treatment (Fig. 5A, lane 5). Accordingly, an ELF-1 expression vector was transfected into HeLa S3 cells in increasing amounts, followed by RT-PCR analysis (Fig. 5C). Levels of *OAS1* transcripts were substantially increased with ectopic expression of ELF-1, when compared to vector only transfected cells.

Western blot analysis indicated a 55 kDa band that was increased with increasing amount of transfection with ELF-1 expression plasmid (Fig. 5D), which is likely to be unmodified ELF-1 protein, whereas the previously reported 90 and 100 kDa bands^23^, which may represent phosphorylated and *O*-linked glycosylated ELF-1 are relatively unchanged. Induction of this faster migrating ELF-1 band was observed in HeLa S3 at 0.5 h after IFN treatment (Fig. 5B). ELF-1 was found to be predominantly nuclear with no apparent changes in sub-cellular localization following IFN treatment (data not shown). Santa Cruz C-20 is an affinity purified rabbit polyclonal antibody raised against a peptide mapping at the C-terminus of ELF-1, suggesting that the C-terminal region contains functional domain. Additionally, shRNA mediated knockdown of ELF-1 was carried out with three target sequences (Fig. 5E; Supplementary table). Knockdown of ELF-1 resulted in reduced expression of *OAS1* when compared to control vector-transfected cells (Fig. 5E). Collectively, ELF-1, whose expression is itself stimulated by IFN, may enhance rapid transcriptional activation of the *OAS1* gene and protein levels in response to IFN.

### ELF-1 enhances IFN-mediated *OAS1* transactivation

**Figure 6:**
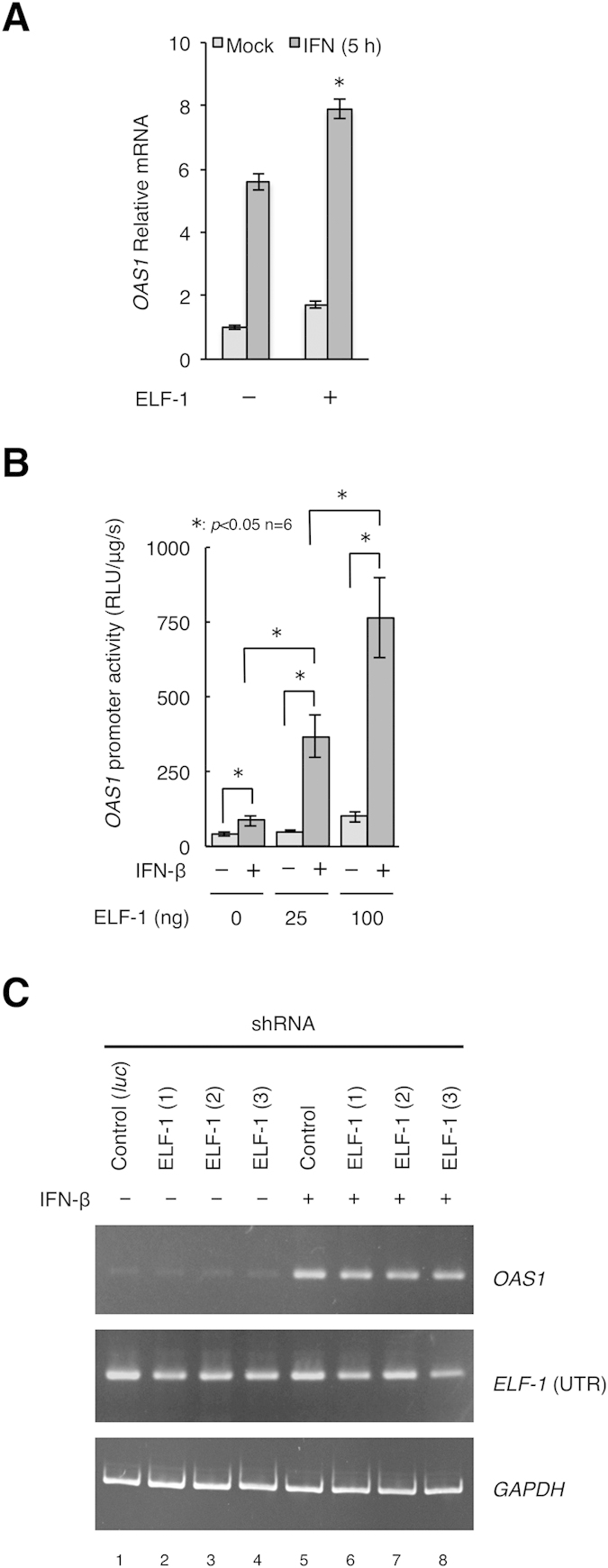
Regulatory function of ELF-1 in initiating IFN-mediated *OAS1* promoter activation and gene expression. (**A**) Quantitative real-time PCR analysis of ELF-1 overexpression in HeLa S3 cells and the effect on IFN-mediated induction of *OAS1* gene expression by 20 ng/ml IFN-β treatment for 5 h. Error bars indicate SD and statistical significance (*p < 0.05) was calculated by the student’s t-test (n = 3). (**B**) Luciferase gene reporter assay showing the effect of overexpression of pcDNA3.1_ELF-1 on IFN-mediated activation of the *OAS1* promoter. Co-transfection of pGL4_OAS1 with increasing amounts of pcDNA3.1_ELF-1 and either mock treatment or treatment with 20 ng/ml IFN-β for 5 h of HeLa S3 cells. Error bars indicate SD and statistical significance (*p < 0.05) was calculated by the students t-test (n = 6). (**C**) RT-PCR for *OAS1* mRNA transcripts from either mock treated or with 20 ng/ml IFN-β for 24 h HeLa S3 cells transfected with the shRNAs in Fig. 5E, results are representative of 3 replicates.

Next the contribution of ELF-1 to *OAS1* gene expression following IFN-stimulation of cells was addressed. When ELF-1 was overexpressed and then the cells were treated with IFN-β for 5 h, *OAS1* gene expression was enhanced (Fig. 6A). Correspondingly, ELF-1 overexpression further enhanced IFN-induced *OAS1* promoter activity in HeLa S3 cells in a dose-dependent manner (Fig. 6B) as determined by luciferase assay. Moreover, in ELF-1 knockdown cells diminished IFN-mediated activation of *OAS1* expression in IFN-β stimulated cells was observed, to approximately half the level of control shRNA transfected cells (Fig. 6C). Taken together these data indicate a functional role for ELF-1 at the *OAS1* promoter in its response to IFN stimulation.

### IFN-β induces RB1 and SP1 recruitment to the *OAS1* promoter and ELF-1 protein interaction with under-phosphorylated RB1 protein

**Figure 7:**
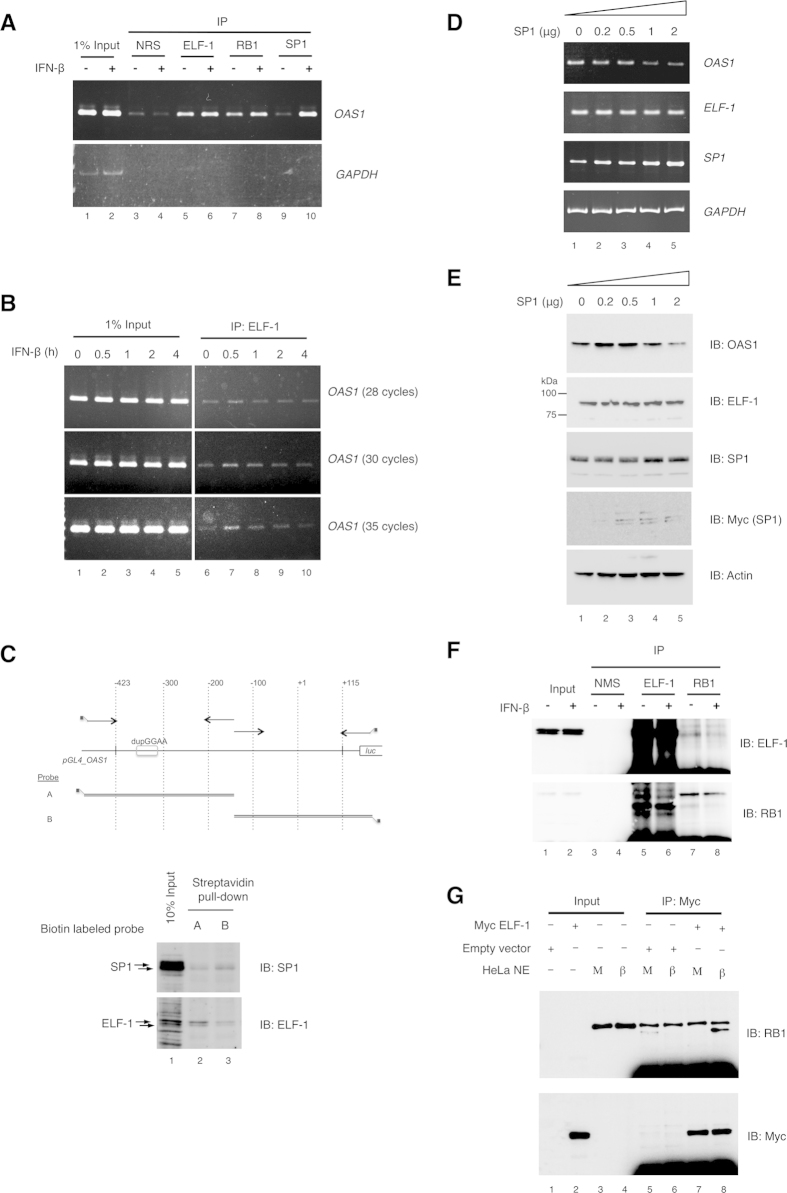
Recruitment of ELF-1, RB1 and SP1 to the promoter region of the *OAS1* gene. (**A**) ChIP assay was carried out with sheared chromatin from Jurkat cells treated with either IFN-β (+) or mock (−) for 2 h, using antibodies targeting ELF-1, RB1, SP1 or normal rabbit serum (NRS). Co-immunoprecipitated DNAs were amplified by PCR with hOAS1(−387) and hOAS1(+3) primers targeting a 390 bp region of the human *OAS1* promoter. Data are representative of 3 experiments. (**B**) Cells were treated with IFN-β for 0, 0.5, 1, 2, and 4 h and ChIP assay was carried out with an ELF-1 targeting antibody (lanes 6–10). Three independent PCR conditions with 28, 30 and 35 cycles were examined. (**C**) DNA-affinity assay, biotin labelled probes A and B derived from sequences in the *OAS1* promoter (upper panel), were incubated with IFN-β treated (5 h) HeLa S3 nuclear extract and the DNA-protein complexes purified by Streptavidin beads. The affinity purified nuclear proteins were analysed by Western blotting with anti-SP1 and anti-ELF1 antibodies (lower panel). (**D**) RT-PCR and (**E**) Western blot analysis of *OAS1*, *ELF-1*, SP1 and *GAPDH* mRNA transcripts and OAS1, ELF-1 and actin protein levels, from HeLa S3 cells after ectopic expression of SP1. HeLa S3 cells were transfected with increasing amounts pcDNA3.1_SP1 expression plasmid or empty pcDNA3.1 plasmid for 24 hours. PCR amplification of cDNA was carried out with the indicated primer sets (Supplementary Table). (**F**) RB1/ELF-1 interaction following IFN-β stimulation. HeLa S3 cells were treated with either mock (−) or IFN-β (+) treated for 5 h, then subjected to co-immunoprecipitation with ELF-1, RB1 antibodies or normal mouse serum (NMS). The co-immunoprecipitated proteins were analyzed by Western blot. (**G**) Immunoprecipitation of proteins from Hela S3 nuclear extracts with *in vitro* translated Myc-tagged ELF-1. The binding reaction included *in vitro* transcription/translation reaction product from either empty pcDNA3.1Myc/his or pcDNA3.1Myc/his_ELF-1, and nuclear extract prepared from HeLa S3 cells mock treated or IFN-β treated (5 h). ELF-1 containing complexes were co-immunoprecipitated with c-Myc antibody for 2 h at 4 °C, and then detection of ELF-1 and RB1 by Western blot.

As an ability of ELF-1 to form complexes with both SP1 and RB1 was previously demonstrated^24^,^30^, we therefore investigated the potential for these proteins to co-occupy with ELF-1 at the *OAS1* promoter. Recruitment of ELF-1 to the *OAS1* promoter was investigated by chromatin immunoprecipitation (ChIP) with antibodies targeting ELF-1, RB1 and SP1, followed by PCR analysis (Fig. 7A). As shown in lanes 5 and 7 respectively, ELF-1 and RB1 occupy a 390 bp region of the *OAS1* promoter in mock treated cells, whereas very low occupation was observed by SP1 (lane 9). Strikingly, IFN-β treatment (2 h) resulted in increased recruitment of not only ELF-1 but also SP1 and RB1 to the region containing GGAA duplication (Fig. 7A, lanes 6, 8 and 10). Time-dependent recruitment of ELF-1 to the *OAS1* promoter was then assessed by ChIP assay following IFN-β treatment (Fig. 7B). Immediate-early recruitment of ELF-1 was observed, peaking at the 30 min time point (Fig. 7B, lane 7) and gradually decreased over time. Using a DNA-affinity assay, we further observed that ELF-1 from IFN-β treated nuclear extracts can bind to the region containing the dupGGAA motif but also that SP1 can physically bind to the region immediately upstream of the *OAS1* transcription start site (Fig. 7C). SP1 was found to bind in the region −157 to +115 nt, computational transcription factor binding site search did not reveal any putative SP1 binding sites. The biotin pulldown assay in Fig. 7C, shows a clear increase in pulldown of ELF-1 protein with probe A (which contains a known ELF-1 binding site) as compared to probe B (no known ELF-1 sites). However, it is not clear whether the fainter band seen for probe B represents the background level or additional lower affinity sites. Additionally, considering that ELF-1 and SP1 have been reported to physically interact, it is possible that indirect binding of these transcription factors was detected. Conversely, overexpression of SP1 in HeLa S3 cells repressed *OAS1* transcription (Fig. 7D,E).

Subsequently we examined changes in the physical interactions between ELF-1 and RB1. As shown in Fig. 7F, co-precipitation of endogenous RB1 with ELF-1 was reduced in cell lysates from IFN treated cells (upper panel, lanes 7 and 8; lower panel lanes 5 and 6), demonstrating an IFN-dependent decrease in the ELF-1/RB1 interaction. Correspondingly, an *in vitro* pull-down with Myc-tagged ELF-1 demonstrated that under-phosphorylated RB1 was pulled down from HeLa S3 nuclear extracts when the cells were treated with IFN-β prior to extraction (Fig. 7G, lanes 7 and 8). Even though under-phosphorylated RB1 appeared to represent a small fraction of the total RB1 (undetectable in the input samples), this faster migrating band was seen with the addition of *in vitro* translated ELF-1 with HeLa S3 nuclear extract from IFN treated cells (compare lanes 7 and 8).

## Discussion

Using transcriptional regulation of ISGs as a starting point, we have demonstrated that a specific DNA-binding complex(s) containing ELF-1 is co-recruited with RB1 and SP1 to a duplicated GGAA motif in the promoter of *OAS1*, one of the main IFN effector genes. We have demonstrated that disruption of a duplicated GGAA containing sequence approximately 300 bp from the *OAS1* TSS resulted in a dramatic reduction of promoter activity. Although limited IFN response was retained, this remaining activity is presumably because of an intact ISRE, without which no activation can be observed^27^. Our data indicate the role of ELF-1 in *OAS1* transcription is not a simple rate-limiting step, such as regulation by the ISRE. Similarly, knockdown of ELF-1 in IFN-stimulated cells also reduced the magnitude of IFN-mediated *OAS1* induction. Such results are indicative of the duplicated GGAA motif constituting a proximal promoter, which exhibit low levels of transcription by themselves but are essential for the activity of enhancers and often function to loop distal regulatory regions to close proximity of the TSS^31^. The duplicity of GGAA motifs is a common occurrence in the promoter region of genes and the specificity of Ets binding factors to a particular GGAA-containing motif for each Ets family protein has been shown elsewhere^32^. Additionally, transcription factor binding sites often occur in close proximity to one another. A wide range of transcription factors could potentially bind sites containing a GGAA-motif. So it could be expected that additional unidentified factors can also bind this motif in addition to ELF-1, either co-operating or competing with ELF-1 depending on the cellular context. Our data show that only the second GGAA motif binds directly to ELF-1. Our results suggest that ELF-1 functions as an important adaptor molecule in modulation of the *OAS1* transcriptional response to IFN stimulation.

GGAA motifs are frequently found in close proximity to the TSS of the majority of ISGs and may prove to be alternative sites for the initiation of transcription^33^, often overlapping with other transcription factor binding sites that helps coordinate gene regulation^34^. Regions containing multiple ETS-binding sites (GGAA) have been shown to be critical *cis*-acting sequences in gene regulation^35^,^36^. Our experiments clearly show that ELF-1 can specifically bind the *OAS1* promoter sequence *in vitro* and endogenous chromatin. However, the mechanism and role of ELF-1 may not be simply defined as a rate-limiting transactivation event, more than likely ELF-1 has a regulatory function. As pre-exposure to some specific cytokines dramatically affects how cells then respond to IFNs^37^, changes to constituents of the IFN-pathway should alter ISG activation patterns. Knockdown experiments suggested that ELF-1 has a positive effect on *OAS1* regulation, affecting the degree of IFN response. Previous studies have reported unchanged mRNA and protein levels of ELF-1 in resting and stimulated T-cells^38^. However, in HeLa S3 cells a rapid (30 min) and transient induction of ELF-1 in HeLa S3 cells stimulated with IFN-β was observed, with no sustained up-regulation of ELF-1 protein levels. Raising the possibility that additional transcription factors are co-recruited to the *OAS1* promoter by physically interacting with ELF-1.

Transcription factor binding to ETS-binding sites and paired co-activator sites may constitute proximal promoter elements in at least some of the 5′ flanking regions of ISGs. We found recruitment of RB1 and SP1 to the region containing the duplicated GGAA motif in the *OAS1* promoter, despite the lack of putative binding sites. Physical interactions of ELF-1 with RB1 or SP1^24^,^30^ may emerge as promising drug targets for precise modulation of the IFN response. RB1 is a well characterized tumor suppressor^39^,^40^ and its loss of function is found in a large proportion of human tumors^41^. The NH_2_-terminal of ELF-1 (amino acids 21–72) binds the pocket domain of RB1. Interestingly, hyper-phosphorylation of RB1 disrupts complex formation demonstrated by overexpression of a phosphorylation-defective RB1 protein^24^. We have found that an ELF-1/underphosphorylated RB1 interaction is induced by IFN-stimulation. The cooperation between ELF-1 and RB1 in IFN stimulated cells suggests that ELF-1 recruits under-phosphorylated RB1 to the *OAS1* promoter upon IFN stimulation. Overexpression of only SP1 had a moderate repressive effect on *OAS1* gene expression in unstimulated cells, even though normal SP1 levels in HeLa S3 cells are high, the exogenously expressed SP1 is likely to be relatively unmodified with respects to post-translational modifications. This suggests that other co-factors such as ELF-1, in addition to SP1 may be needed to counteract the repressive effect of SP1 following IFN stimulation.

The classical JAK-STAT signal transduction pathway alone is not sufficient for generation of the full range of IFN-mediated effects. Instead, various signaling pathways mediate the biological functions of IFNs^42^. Differential responses of cell subtypes to IFNs are reported^9^,^10^, consistent with this and our present findings, ELF-1 may be involved in modulation of immune responses of particular cell types. The evidence presented here implies that recruitment of ELF-1 to the *OAS1* promoter enhances the response to IFNs. The appropriate response to IFNs (dependent on cell type and existing cell state) might be determined by the availability of ELF-1 for transcriptional activation, competition with other GGAA-binding factors, post-translational modifications and changes in composition of ELF-1 complexes. Clearly, ELF-1 can occupy the *OAS1* promoter in unstimulated cells, but a small increase in occupation was also observed following IFN stimulation. Taken together, these data permit speculation that an increase in cellular ELF-1 levels allows for a faster or greater IFN response because of this pre-occupancy. A possible mechanism for this is that in non-stimulated cells, either an ELF-1 or an ELF-1/SP1 complex and hyper-phosphorylated RB1, are dissociated or loosely associated with the *OAS1* promoter and only basal transcription levels are observed. As ELF-1 and SP1 can physically interact in the absence of DNA, we envision that upon IFN-stimulation de-phosphorylation of RB1 protein occurs, which then permits RB1 to form a complex with ELF-1/SP1. Such mechanisms could at least partly account for why particular cells of the immune system respond to the exact same extrinsic signals in very different ways. Explicitly, the extracellular IFN signal is very broad and it is the capacity and availability of specific cellular factors, such as receptors, signaling molecules and transcription factors that determine IFN-mediated cellular responses. Therefore, future work is required to investigate whether ELF-1 is an additional key molecule in the IFN signal transduction pathway, acting as a general or specific transcriptional regulator of ISG expression. Our findings suggest that in addition to the essential JAK/STAT activation of ISGs, ELF-1 as well as other GGAA motif-binding factors should be taken into account when studying IFN-mediated cellular responses. Therefore, ELF-1 mediated regulation of *OAS1* may represent one way in which cell type specific differences in IFN-responses could occur. That is, prevailing expression levels of transcription factors predispose the cell to a particular ISG expression pattern and specific IFN-response.

Obtaining a deeper understanding and further insights into the basic mechanisms required for effective gene expression in response to IFN will enable us to rationally design innovative therapeutic agents. IFN therapy is currently used for the treatment of some cancers, viral infections and immune diseases but the serious side effects^43^,^44^,^45^ limit the number and spectrum of patients that may benefit from it. Although IFN-β therapy in multiple sclerosis is hailed as a breakthrough^12^, it is only partially effective and therefore greater advances on IFN therapy are required^13^. Recent reports have suggested a link between single nucleotide polymorphisms (SNPs) in the *OAS1* gene that strongly contribute to the prognosis of the disease^46^, implying that OAS1 may play a fundamental role in the ability of IFNs to treat multiple sclerosis. GGAA-containing motifs and host ELF-1 have a demonstrated role in the gene transcription of the Human Immunodeficiency Virus 2^47^. Interestingly, ELF-1 has been identified as a cellular target of Epstein Barr Virus, which is associated with Burkitt’s lymphoma^48^. ELF-1 is a predominant cellular factor that binds to the enhancer of the human T-cell leukemia virus type 1 (HTLV-1), the etiological agent of T-cell lymphoma/leukemia. Mutation analysis of this site markedly reduced inducible activity of the HTLV-1 enhancer^49^. Exploratory studies are required to understand the role of ELF-1 in anti-viral immunity, at present we speculate that targeting of ELF-1 in virus related diseases maybe useful as some viruses may have either evolved dependence on, or impede ELF-1 function. To conclude, understanding the action of IFN-β on the expression of *OAS1* and other ISGs may help establish alternative and enhanced therapies, making IFN-derived treatments available for a wider spectrum of patients and diseases. This could be achieved by mimicking distinct IFN effects, for example by gene-therapy or small molecule compounds targeting protein-protein interactions in diseases where previously only treatment with IFNs has been effective.

Here we have identified sequence dependent binding of the ELF-1 transcription factor and a role in transactivation of the *OAS1* promoter. We have provided additional supporting data that suggest the ELF-1 interacting partners, SP1 and RB1 proteins are also recruited to the *OAS1* promoter. We are currently investigating in full the dynamics and contributions of SP1 and RB1 with ELF-1 in activation/regulation of Interferon stimulated genes.

## Methods

### Bioinformatic analysis

For occurrences of duplicated GGAA motifs, genomic sequences −500 to +100 bp surrounding the putative TSS of ISGs were retrieved from the Ensembl database using the web-based Regulatory Sequence Analysis Tools (RSA-Tools; http://rsat.ulb.ac.be/)^50^,^51^,^52^. The DNA pattern search tool was utilized to search for the presence of the duplicated GGAA motifs with the following string descriptions: GGAAN{0,10}GGAA and GGAAN{0,10}TTCC; where N{0,10} indicates a spacer of between 0 and 10 nucleotides, results for both sense and anti-sense strands were retrieved. The results were then displayed graphically using the RSAT feature map tool. For comparison, two sets of control sequences were acquired, one of humanized random DNA sequences of 600 bp; and the second from −500 to +100 bp of the TSSs for the promoters of randomly selected human genes.

### Cell lines, transfection and Luciferase assays

Jurkat and HL-60 cell lines cultured in RPMI 1640 medium and HeLa S3 cells in DMEM, were maintained at 37 °C/5% CO_2_. Culture media was supplemented with 10% FBS, 100 IU/ml penicillin and 100 μg/ml streptomycin. For overexpression experiments, cells were transfected with expression plasmids using XtremeGene 9 transfection reagent (Roche Applied Science, Indianapolis, IN) according to the manufacturers’ recommendations. Total amount of transfected plasmid DNA was adjusted to constant levels with empty parental vector. For multi well *Luc* reporter assays in 96-well plates, a DEAE-dextran based transfection method was used, as described previously^53^, cell lysate was prepared with 1× cell culture lysis reagent and subjected to *Luc* assay (Promega, Madison, IN).

### Construction of plasmids

The ELF-1 and SP1 open reading frames (ORF) and was amplified by PCR with primers, (Supplementary Table), containing KpnI and XhoI restriction sites with template cDNAs from HL-60 cells, and ligated into the multi-cloning site of pcDNA3.1Myc/His-B mammalian expression vector (Invitrogen, Carlsbad, CA). Sequencing of the *ELF-1* ORF identified two SNPs in HL-60 derived ELF-1, which are reported in the NCBI dbSNP database (rs7799 and rs1056820). The resultant plasmids were designated, pcDNA3.1_ELF-1 and pcDNA3.1_SP1. pGL4 *Luc* reporter vectors (Promega) carrying the human *OAS1* promoter and its deletions were constructed in the manner previously described^53^. Mutations were introduced using site directed mutagenesis of the pGL4_OAS1 plasmid based on^54^. Briefly, PCR was performed with opposing primers (Supplementary Table) containing the desired mutation and the entire plasmid was amplified using Phusion polymerase (Finnzymes oy, Vantaa, Finland) in high fidelity (HF) buffer, the parental hemi-methylated strand was digested with DpnI before being used for transformation of *E. Coli*. All plasmid constructs were confirmed by sequencing (FASMAC, Yokohama, Japan).

### Reverse transcription-PCR (RT-PCR) and real-time quantitative PCR

Total RNAs were isolated from cells using Isogen (Nippon Gene, Tokyo, Japan). The RNAs were quantified and equivalent amounts subjected to reverse transcription with ReverTra Ace (Toyobo, Tokyo, Japan), the resultant first strand cDNA was subjected to PCR using the gene specific primer sets (Supplementary Table) as indicated in figure legends. Amplification of *GADPH* cDNA was used as an internal control. For analysis of mRNA transcripts in shRNA-mediated knockdown experiments, real-time quantitative PCR was carried out using SYBR green real time PCR mix (Toyobo) and analyzed on ABI 7300 real-time PCR machine (Applied Biosystems). The efficiency of the primers in the PCR reactions was checked against a standard curve using serially diluted pooled cDNA. All reactions were performed in triplicate and normalized to *GAPDH* expression levels, calculations were performed using pyQPCR analysis suite (http://pyqpcr.sourceforge.net/) by the ΔΔCt method.

### shRNA mediated gene knockdown

pGeneClip U1 hairpin expression vectors (Promega) containing either control (luciferase) or ELF-1 targeting shRNAs under the control of the U1 promoter were prepared following the manufacturers instructions. Transfected HeLa S3 cells were selected with Hygromycin B for 2 weeks before either mock or IFN treatment. Sequences for shRNA templates are shown in the Supplementary Table.

### Electrophoretic-mobility shift assay (EMSA) and DNA-affinity purification

Nuclear extracts were prepared from either mock or IFN treated cells essentially as previously described^55^. Duplex DNA probes were created by annealing partially complementary oligos, filling in the ends with Klenow fragment (Toyobo), and then end-labeled with digoxigenin (DIG) (Roche Applied Science), the sequences for duplex DNA probes and competitors are shown in Table 2. Binding reactions were carried out in; 0.2 mM EDTA, 20% Glycerol, 20 mM Hepes-KOH (pH7.9), 100 mM KCl, 1 mM DTT, 1 mM PMSF; for 15 min at 20 °C, the resulting reaction mixture was separated by native TBE-PAGE and transferred to a positively charged nylon membrane (PALL, Port Washington, NY) in 0.5× TBE buffer and UV cross-linked with a transilluminator. Detection of labeled DNAs was performed with an alkaline phosphatase conjugated anti-DIG antibody and CSPD ECL substrate (Roche Applied Science); chemiluminescence was detected using LAS 4000 imager (Fuji Film, Tokyo, Japan). For competition-EMSAs, a molar excess of unlabeled competitor probe was included in the binding reaction, as indicated in figure legends. *In vitro* transcription and translation of ELF-1 was carried out using the TNT quick coupled transcription/translation system (Promega), 3 μl of translated protein was substituted for the nuclear extract where indicated. *In vitro* translated ELF-1 was confirmed by Western blot. Supershift-EMSAs were performed by incubating the binding reaction with the indicated antibody for an additional 45 min at 20 °C. For biotin-mediated DNA-affinity purification and detection of ELF-1 and SP1 by western blotting, 3′-end biotin labeled dsDNA probes were prepared by incorporating biotinylated primers (Supplementary Table) during PCR amplification of the OAS1_pGL4 plasmid, followed by cleanup with a PCR purification kit (NipponGenetics). Biotinylated probes were bound to streptavidin beads, washed twice in EMSA binding buffer and then incubated with 32 μg IFN-β treated HeLa S3 nuclear extract in the presence of 1 μg poly dI:dC in 200 μl of EMSA binding buffer for 50 min at 20 °C. The beads were then washed 3× with binding buffer containing 0.1% Tween-20 and bound proteins were eluted by boiling in 2× SDS sample buffer before being separated by SDS-PAGE and detected by Western blot analysis.

### Immunoprecipitation and Western blot analysis

Western blot and immunoprecipitation were essentially carried out as described previously^56^. Briefly, cell lysates were separated by denaturing SDS-PAGE, transferred to Protran nitrocellulose membrane (Millipore, Billerica, MA) and immunoblotted with the following primary antibodies targeting RB1, ELF-1, Myc and an β-actin (Santa Cruz Biotechnology, Santa Cruz, CA) proteins, followed by secondary reaction with either horse radish peroxidase (HRP) or alkaline phosphatase (AP) conjugated anti-mouse or -rabbit IgGs (Calbiochem, Billerica, MA). HRP signals were detected by enhanced ECL substrate (Perkin Elmer, Boston, MA) and AP signals with BCIP/NBT (Sigma-Aldrich, St. Louis, MO). For preparation of nuclear and cytoplasmic cell extracts, fractions were prepared by lysing the cell membrane in 700 μl buffer per 5 × 10^6^ cells containing; 10 mM Tris.HCl (pH 7.5), 1 mM EDTA, 0.5% NP-40 and proteinase inhibitor cocktail (Sigma-Aldrich) on ice for 10 min, centrifugation at 10 000 × g and the supernatant (cytoplasmic extract) was recovered, the nuclear pellet was washed twice in lysis buffer and lysed in SDS-sample buffer. For immunoprecipitation of proteins from HeLa S3 nuclear extracts with *in vitro* translated Myc tagged ELF-1. The reaction included 10 μl of TNT quick coupled *in vitro* transcription/translation (Promega) reaction product with either empty pcDNA3.1Myc/his or pcDNA3.1Myc/his_ELF-1 as a template, 320 μg nuclear extract prepared from HeLa S3 cells mock treated or IFN-β treated, in PBS to a final volume of 500 μl, 1 mM DTT and 1× proteinase inhibitor cocktail (Roche Applied Science), incubated for 1 h at 4 °C. The ELF-1 containing complexes were immunoprecipitated with 2 μg c-Myc antibody for 2 h at 4 °C, recovered by protein G sepharose beads and separated on a SDS-PAGE, followed by Western blot analysis.

### Chromatin Immunoprecipitation (ChIP) assay

ChIP assay was performed as in the EZ-ChIP protocol (Millipore); to reduce background, magnetic Dynabeads (Invitrogen) replaced protein G agarose. Briefly, Jurkat cells were treated with 20 ng/ml IFN-β for 0–4 h, cross-linked with 1% formaldehyde and chromatin sheared by sonication (200–1000 bp fragments), then immunoprecipitated with antibodies targeting ELF-1, RB1 and SP1 (Santa Cruz Biotechnology). Enriched DNAs were amplified by PCR with promoter specific primer sets (Supplementary Table). A primer set for the *GAPDH* control region was provided in the EZ-ChIP assay kit (Millipore).

### Non-radioisotope *in vitro* transcription and run-off assay (NITRA)

IFN-β treated HeLa S3 nuclear extract, was pre-incubated for 15 min on ice with 150 fmol linearized template (SpeI/PvuII fragments of basic pGL4[*luc*2.10], pGL4_OAS1Δ1 or pGL4_mtdupGGAA), in a 20 μl reaction containing, 0.5× binding buffer, 7.5 mM MgCl_2_, and 20 U RNAse inhibitor (Toyobo). The reaction was initiated by addition of 0.8 μl of 10 mM rNTP mix containing DIG-11-UTP (Roche Applied Science) to a final concentration of 0.4 mM ATP, 0.4 mM GTP, 0.4 mM CTP, and 0.26 mM DIG-11-UTP. Then incubated at 30 °C for 60 min, stopped with 180 μl stop buffer (10 mM EDTA, 0.2% SDS, 0.3 M sodium acetate, 50 μg/ml yeast tRNA) and RNA-bound proteins digested with 10 μg of proteinase K for 15 min at 55 °C. *De novo* RNA transcripts were purified by extraction with TE-saturated phenol and then phenol/chloroform, followed by ethanol precipitation. Then, separated by 8 M Urea denaturing PAGE, electrophoretically transferred to a nylon membrane in TBE and cross-linked to the membrane by UV-irradiation. DIG-labeled RNAs were detected with DIG-detection reagents (Roche Applied Science).

## Additional Information

**How to cite this article**: Larsen, S. *et al.* The hematopoietic regulator, ELF-1, enhances the transcriptional response to Interferon-β of the *OAS1* anti-viral gene. *Sci. Rep.* **5**, 17497; doi: 10.1038/srep17497 (2015).

## Supplementary Material

Supplementary Information

## Figures and Tables

**Table 1:** Frequency of duplicated GGAA motifs in the promoters of human ISGs.

	% ISG	% Randomly selected gene	% Humanized random DNA sequence
Duplicated GGAA motif^a^	81 (47)	51.7 (30)	25 (15)
No duplicated GGAA motif	19 (11)	48.3 (28)	75 (45)
Total	100 (58)	100 (58)	100 (60)

^a^Results indicate the % occurrence of a duplicated GGAA motif defined by the following string descriptions: GGAAN{0,10} and GGAAN{0,10}TTCC; the number of retrieved sequences is displayed in parenthesis.

**Table 2:** dsDNA duplex probes and competitors used for EMSA.

Duplex DNA	Sequence^a^
OAS1 −340/−301	5′-TTAGAATCATAGATGATCTTTCCACTTCCTGGTTTTTCTG-3′
OAS1 −379/−331 (Non-specific)	5′-AGCTCTATATCAAAACGTTTATAGAGGCAATTTTGTAGTGTTAGAATCAT-3′
OAS1 −330/−301	5′-AGATGATCTTTCCACTTCCTGGTTTTTCTG-3′
mtTTCC1	5′-AGATGATCTAAGGACTTCCTGGTTTTTCTG-3′
mtTTCC2	5′-AGATGATCTTTCCACAAGGTGGTTTTTCTG-3′
Duplicated GGAA motif	5′-GAGCTCGCTAGCCTCGAGGGAAGCGGGAACCGCCGAGGCCAGATCT-3′

^a^*GGAA (TTCC) motifs* are underlined. Only sense strand is shown.
