# Supplementary material for: The hematopoietic regulator, ELF-1, enhances the transcriptional response to Interferon-β of the OAS1 anti-viral gene
Source: Sci Rep. 2015 Dec 8;5:17497. doi: 10.1038/srep17497 (PMC4672336; doi:10.1038/srep17497)
Supplement: Supplementary Information [file srep17497-s1.pdf]

The hematopoietic regulator, ELF-1, enhances the transcriptional response to Interferon-β of the *OAS1* anti-viral gene

Steven Larsen, Shota Kawamoto, Sei-ichi Tanuma and Fumiaki Uchiumi

Supplementary Table Primers and oligos used in this study

Use	Target	Sense 5' to 3'	Antisense 5' to 3'
Promoter analysis	wtOAS1	hOASL: TCGGTACCTGTGTTTGTGTGTGTGTTAAC	ahOASL: ATCTCGAGATCCATCATTGACAGGAGAGAG
	OAS1Δ1	D1hOASL: CAGGTACCGCTCTATATCAAAACGTT	ahOASL: ATCTCGAGATCCATCATTGACAGGAGAGAG
	OAS1Δ2	D2hOASL: CAGGTACCGGCAGTGGGCATGTATTG	ahOASL: ATCTCGAGATCCATCATTGACAGGAGAGAG
	OAS1Δ3	D3hOASL: CAGGTACCGACACAAAATCTGTTAAA	ahOASL: ATCTCGAGATCCATCATTGACAGGAGAGAG
Site directed mutagenesis	OAS1	s_OAS1_SDM1: AGATGATCTTCTCACTCTCTGGTTTTTCTG	as_OAS1_SDM1: CAGAAAAACCAGAGAGTGAGAAGATCATCT
Expression plasmid	ELF-1 ORF	s_ELF1_CDS_KpnI: GCAGGTACCATTATGGCTGCTGTTGTCC	as_ELF1_CDS_xhoI: GCACTCGAGCTACTAAAAAGAGTTGGGT
ChIP	OAS1 promoter	hOAS1(-387): TCTGGAAAGCTCTATATCAAAACG	hOAS1(+3): GGAGGAGCTGTCTTTGCACT
RT-PCR	OAS1	OAS1_741_765: TCAAGCACTGGTACCAAAATTGTAA	asOAS1_1185_1166: CTGGGATCGTCGGTCTCATC
	ELF-1 5' UTR	ELF1_52_71: TCCCCAATCTACAGGAGCCA	asELF1_460_411: AGCTGGATCACCAAGCTGTC
	ELF-1 ORF	s_ELF1_CDS_KpnI: GCAGGTACCATTATGGCTGCTGTTGTCC	as_ELF1_CDS_xhoI: GCACTCGAGCTACTAAAAAGAGTTGGGT
	GAPDH	hGAPDH556: TGCACCACCAACTGCTTAGC	hGAPDH642: GGCATGGACTGTGGTCATGAG
Real-time PCR	OAS1	hOAS1_1037RT: GGAGACCCAAAGGGTTGGAG	as_hOAS1_1154RT: TGCTTTCAGCCAGCAGAATC
	ELF-1	hELF1_619RT: CCTTACAGTTGAAGCTTCTTGTCAT	as_hELF1_780RT: GGGGCAACAACCATGTCATC
	GAPDH	hGAPDH556: TGCACCACCAACTGCTTAGC	hGAPDH642: GGCATGGACTGTGGTCATGAG
shRNA target sequences	Luciferase	GCTGAGTACTTCGAAATGTCCTTCCTGTCAGACATTTCGAAGTACTCAGC	
	ELF-1 (1)	GAAGAGCCCAATGACATGATCTTCCTGTCAATCATGTCATTGGGCTCTTC	
	ELF-1 (2)	GAATTATGAGACCATGGGAACTTCCTGTCATTCCCATGGTCTCATAATTC	
	ELF-1 (3)	GAAGCAGCTAGAACCAGTACCTTCCTGTCAGTACTGGTTCTAGCTGCTTC	

KpnI (sense), *XhoI* (anti-sense) restriction sites are underlined.
